# Polyclonal Antibody Generation against PvTRAg for the Development of a Diagnostic Assay for *Plasmodium vivax*

**DOI:** 10.3390/diagnostics13050835

**Published:** 2023-02-22

**Authors:** Shalini Aggarwal, Selvamano Selvaraj, Jayaprakash Nattamai Subramanian, Mookambeswaran Arunachalam Vijayalakshmi, Swati Patankar, Sanjeeva Srivastava

**Affiliations:** 1 Department of Biosciences and Bioengineering, Indian Institute of Technology Bombay, Mumbai 400076, India; 2 Department of Molecular Genetics, Weizmann Institute of Science, Rehovot 7610010, Israel; 3 Centre for Bio-Separation Technology, Vellore Institute of Technology, Vellore 632014, India

**Keywords:** *Plasmodium vivax*, *Plasmodium* species, malaria, diagnosis, PvTRAg

## Abstract

The World Health Organization (WHO) has set forth a global call for eradicating malaria, caused majorly by the protozoan parasites *Plasmodium falciparum* and *Plasmodium vivax*. The lack of diagnostic biomarkers for *P. vivax*, especially those that differentiate the parasite from *P. falciparum*, significantly hinders *P. vivax* elimination. Here, we show that *P. vivax* tryptophan-rich antigen (PvTRAg) can be a diagnostic biomarker for diagnosing *P. vivax* in malaria patients. We report that polyclonal antibodies against purified PvTRAg protein show interactions with purified PvTRAg and native PvTRAg using Western blots and indirect enzyme-linked immunosorbent assay (ELISA). We also developed an antibody-antigen-based qualitative assay using biolayer interferometry (BLI) to detect vivax infection using plasma samples from patients with different febrile diseases and healthy controls. The polyclonal anti-PvTRAg antibodies were used to capture free native PvTRAg from the patient plasma samples using BLI, providing a new expansion range to make the assay quick, accurate, sensitive, and high-throughput. The data presented in this report provides a proof of concept for PvTRAg, a new antigen, for developing a diagnostic assay for *P. vivax* identification and differentiation from the rest of the *Plasmodium* species and, at a later stage, translating the BLI assay into affordable, point-of-care formats to make it more accessible.

## 1. Introduction

*Plasmodium vivax* (*P. vivax*) elimination requires early diagnosis leading to efficient treatment. *P. vivax* detection methods include microscopy, rapid diagnostic tests, and nucleic acid amplification [1]. However, each method has its limitations regarding accurate and quick diagnosis. The problem elevates when the parasitemia levels are low, making it difficult for clinicians to detect the parasite accurately. Microscopy is the gold standard for malaria parasite detection and diagnosis but is time-consuming and prone to human error. Additionally, the International Standard (IS) for immunoassays for *P. vivax* is the antigen *P. vivax* lactate dehydrogenase (PvLDH), a protein found in all *Plasmodium* species due to its essential role in metabolism. Therefore, diagnostic tests based on this antigen lack a differentiating protein biomarker to differentiate *P. vivax* from the other *Plasmodium* species [2,3,4]. A protein biomarker candidate, unique to *P. vivax* with little or no identity with other *Plasmodium* species and the host, would be ideal for generating antibodies for immunoassay development. However, such biomarkers have been challenging to identify due to scarcity in parasite availability, either due to low parasitemia in the clinical samples or no continuous culturing of the parasite.

Immunoassay development requires polyclonal antibody development as the first step to confirm the antigenic characteristics of the target antigen. Polyclonal antibodies are a collection of immunoglobulin molecules that recognize different epitopes of a specific antigen. Since these antibodies are directed against multiple regions/epitopes of the antigen, they are called polyclonal antibodies. Consequently, they have different antigen specificities and affinities towards the antigen [5], making them suitable for preliminary screening of candidate antigens for diagnosis. These antibodies can be produced by immunizing suitable mammals such as mice [6], rabbits [7], or goats [8] with an antigen. Injected antigen induces the plasma cells (B-lymphocytes) to create the immunoglobulin, specifically for the antigen. This polyclonal immunoglobulin gamma (IgG) can then be purified from the animal’s serum. The primary goal of mammalian antibody production is to obtain a high titer and high-affinity antisera for diagnostics and other applications [5,9,10]. 

For the last decade, our team has worked on comprehensive parasite proteome analysis, using whole blood samples of vivax-infected Indian patients. Proteome analysis facilitated the search for diagnostic biomarker candidates. Still, due to challenges presented by parasite biology (low parasitemia and no continuous in vitro culturing), extraction of the parasite was challenging. The recent coronavirus disease (COVID-19) pandemic made correct sample collection arduous. In the era of the internet, knowledge sharing is easier, and to increase confidence in the findings of the most recurring parasite proteins, literature-based [11,12,13,14,15,16] data mining was also performed. Five parasite proteins, PVX_094303, PVX_003545, PVX_090265, PVX_101520, and PVX_083555, were found to be recurring in proteomics studies of *P. vivax* clinical samples (Supplementary Figure S1). 

*Plasmodium* has eight pv-fam gene families viz. pv-fam-a, pv-fam-b, pv-fam-c, pv-fam-d, pv-fam-e, pv-fam-g, pv-fam-h, and pv-fam-I [17]. The pv-fam-a family was reported to express and show abundance in *P. vivax* compared to *P. falciparum* [18]. Members of the pv-fam-a gene family have shown high immunogenicity in humans [17,19]. PVX_090265 and PVX_101520 belong to pv-fam; however, PVX_090265 has no identity with the human proteome, making it an ideal biomarker candidate. Hence, in the study, we used purified PvTRAg (PVX_090265) protein to generate the polyclonal antibodies using a five-month-old New Zealand rabbit (approximately 2 kg). The generated polyclonal antibody was purified using protein A-based affinity chromatography. The purified antibody was further used to test and validate the interaction of synthesized polyclonal antibodies against purified PvTRAg and native PvTRAg. It was observed that the polyclonal antibody generated using heterogenous PvTRAg could bind to the native PvTRAg. The purified antibody and antigen were utilized to create a diagnostic assay using vivax-infected patient plasma. Both the experimental strategies were explored for indirect detection of parasites in the plasma samples, i.e., by identifying naturally synthesized antibodies in the host against PvTRAg. Results indicate that PvTRAg is a suitable antigen that could be taken further for diagnosing and differentiating *P. vivax* from pan *Plasmodium* species.

## 2. Materials and Methods

### 2.1. Polyclonal Antibody Generation against PvTRAg for the Development of a Diagnostic Assay for P. vivax

**Figure 1:**
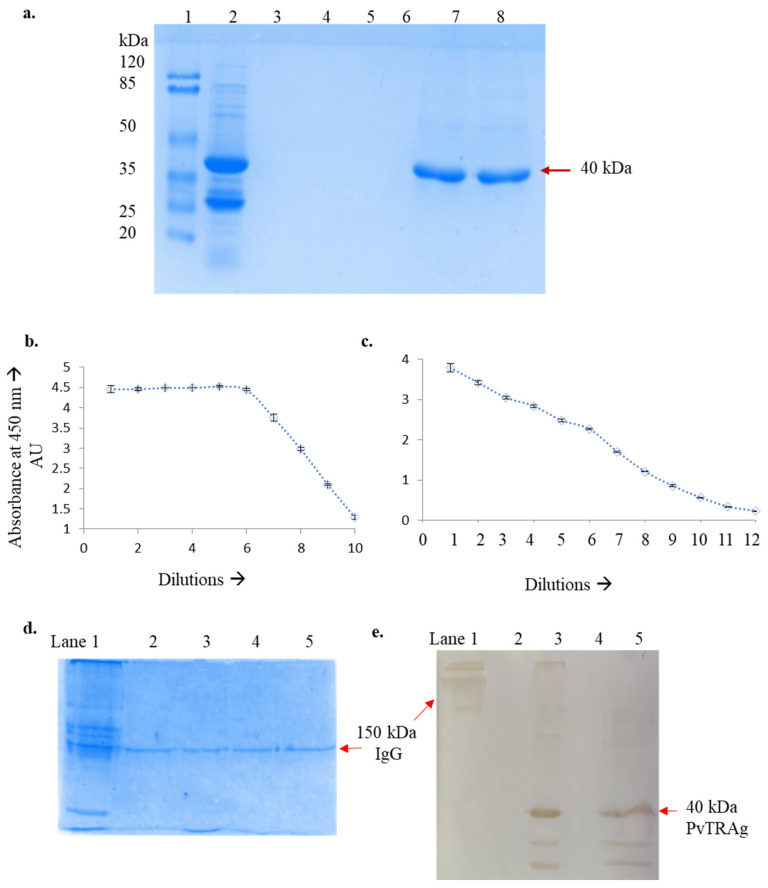
(**a**) SDS-PAGE of PvTRAg protein, lane 1: protein MW marker; lane 2: crude protein sample (solubilized inclusion bodies); lanes 3-6: empty lanes; lanes 7 and 8: gel purified PvTRAg protein. (**b**) Indirect ELISA for first bleed dilutions 1:100 (1 part rabbit sera to 99 parts 1× PBS) to 10 dilutions in a 1:1 ratio (1 part 1st dilution of rabbit sera to 1 part 1× PBS), with 1 µg/well PvTRAg. (**c**) Indirect ELISA for first bleed dilutions 1:1000 (1 part rabbit sera to 999 parts 1× PBS) to 12 dilutions in a 1:1 ratio (1 part 1st dilution of rabbit sera to 1 part 1× PBS), with 1 µg/well PvTRAg. (**d**) The 10% SDS-PAGE profile of the purified polyclonal antibody using protein A affinity chromatography, where lane 1 is crude sera of rabbit and lanes 2 to 5 are elution fractions E6 to E9. (**e**) Western blot image representing the identification of PvTRAg antigen using purified polyclonal antisera of the rabbit, where lane 1 is IgG control, lane 2 is empty, lane 3 is 5 µg purified PvTRAg with non-reducing loading dye, lane 4 is empty, and lane 5 is 5 µg purified PvTRAg with reducing loading dye.

The parasite protein PvTRAg was abundant in the previous studies done by our research group and literature-based data mining [14,20]. The protein was cloned and expressed in *E. coli* BL21 DE3 pLyS, which produced the protein as inclusion bodies. These inclusion bodies were then solubilized using 0.5% N-lauryl sarcosine [21]. The solubilized protein was found to be around 95% pure, and the sodium dodecyl-sulfate polyacrylamide gel electrophoresis (SDS-PAGE) profile is shown in Figure 1a. The protein was aliquoted in 4 parts with 1 mg per vial and stored at −80 °C for further processing. 

### 2.2. Immunization of Rabbits and Titration Optimization

The 1 mg aliquot was taken and dissolved in 400 µL of 1× phosphate-buffered saline (PBS) to make it 2.5 µg/µL, and 250 µg of the purified protein was mixed with 250 µL of complete Freund’s adjuvant (CFA) and injected intradermally into the laboratory-bred female New Zealand albino rabbit [22]. The study was ethically approved by the Vellore Institute of Technology (VIT) ethical committee, VIT/IAEC/20/DEC2021/09. The sensitization of the animal was done using 250 µg of purified PvTRAg mixed with 250 µL of incomplete Freund’s adjuvant for the booster doses at an interval of 21 days. After the booster dose, a test bleed was collected on the 7th day from the marginal ear vein of the animal, and the titer value of antisera was checked using antigen-dependent ELISA.

### 2.3. Rabbit Polyclonal Antibody Titer Check Using Indirect ELISA

ELISA plates (Thermo, Nunc maxisorp, Waltham, MA, USA) were coated with purified PvTRAg (1 µg/well) suspended in 100 µL of 100 mM carbonate-bicarbonate buffer (0.42 g of sodium bicarbonate (NaHCO_3_) and 0.52 g of sodium carbonate (Na_2_CO_3_) in 50 mL Milli-Q water, pH 9.6) and incubated overnight at 4 °C on the static condition. A total of 200 µL of 1× phosphate buffer saline-tris (1× PBST) was added to the plate to remove the excess coating buffer and unbound PvTRAg and incubated for 5 min at room temperature (RT) under static conditions. The plates were washed using 200 µL of 1× PBS, pH 7.2, twice and blocked with 10% skim milk in 1× PBS for 1 h at 37 °C. Four washes of 200 µL of 1× PBST with 5 min incubation were used to remove the unbound blocking buffer. The anti-PvTRAg sera ten dilutions, ranging from 1:100 to 1:51,200 (sera: 1× PBS), were added as primary antibodies and incubated for 1 h at 37 °C under static conditions. The plate was washed four times using 200 µL of 1× PBS to remove non-specific or unbound sera/primary antibodies. The primary antibody was detected using goat-origin anti-rabbit IgG-horseradish peroxidase-conjugated secondary antibody (1:3000, antibody: 1× PBS). We added 100 µL of the secondary antibody diluents to the plate and incubated it for 1 h at 37 °C under static conditions. The plate was washed to remove the excess amount of secondary antibody using 1× PBS with 5 min of incubation four times. We added 20 µL of TMB/H_2_O_2_ to 20 mL of 1× PBS, and 100 µL of the solution was added to each well. The plate was incubated at RT for 8 min in a dark condition. The reaction was stopped by adding 50 µL of 2 M sulfuric acid, and optical density (OD) was measured at 450 nm using a multi-well plate reader (FLUOstar Optima, B.M.G. Labtech, Ortenberg, Germany).

Nine days after the second booster dose, the animal was euthanized, and the whole blood was collected from the jugular vein. The blood was clot at RT for 1 h and then centrifuged at 1500 rpm for 30 min at 4 °C to collect the antisera as the polyclonal antibody.

### 2.4. Purification and Characterization of Rabbit Polyclonal Antibody

The produced anti-PvTRAg, polyclonal antibodies (pAb), were purified to near homogeneity by protein A-sepharose affinity chromatography. A total of 1 mL of protein A-Sepharose bead-packed column was washed using the 5-bed volume of 1× PBS. The 100 µL of rabbit polyclonal antibody or rabbit sera was diluted with 900 µL of 1× PBS and passed through the equilibrated column containing protein A. Protein A has a high affinity for IgG antibodies. The bound antibodies were eluted using 50 mM glycine buffer, pH 2.5. The elute was collected in 500 µL aliquots; the elution buffer has low pH that may degrade the eluted antibody; hence the elution buffer was mixed with 1 M tris base (pH 10) until the pH was in the 7.4 to 7.7 range. The neutralized solution was then quantified using Bradford, and 2.5 µg of each eluted fraction was run on SDS-PAGE for a quality check. The purified antibody was further taken for characterization using Western blot and indirect ELISA.

#### 2.4.1. Western Blot Using Purified PvTRAg and Rabbit Polyclonal Antibody

The purified protein (PvTRAg) was electrophoresed in 12% SDS-PAGE according to the standard method described by Laemmli [23]. The protein on the SDS-PAGE was transferred to the polyvinylidene fluoride (PVDF) membrane using wet blot transfer assembly at 90 V on a constant 359 mA for 90 min. The PVDF membrane was checked for transfer of bands using ponceau stain. The PVDF membrane was submerged in 10 mL of 5% skimmed milk in 1× tris-buffer saline (1× TBS) and incubated on the rocker at 20 rpm at 4 °C overnight to block the PVDF membrane’s unbound surface. The next day the membrane was washed using three rounds of 10 mL 1× tris-buffer saline with 0.5% Tween 20 (1× TBST) on the rocker at 50 rpm. Post-washing, the membrane was subjected to the anti-PvTRAg polyclonal antibody as primary (rabbit polyclonal antibody) in a 1:1000 dilution made in 1× TBST. The membrane was then incubated at RT for two hours on the rocker at 20 rpm. The membrane was washed thrice after incubation using 10 mL of 1× TBST on the rocker at 50 rpm. The bound rabbit polyclonal antibody on PvTRAg was identified using goat antibodies to rabbit IgG conjugated in a 1:3000 dilution, made in 1× TBS as a secondary antibody. The PVDF membrane was incubated at RT for 2 h on the rocker at 20 rpm. The washing step was repeated thrice after incubation using 10 mL of 1× TBST on the rocker at 50 rpm. The washed membrane was developed using 20 mL tris base pH 7.2 with 5 mg diaminobenzylene (DAB) and 3 mg nickel chloride solution mixed with 5 µL tetramethylbenzidine (TMB)/H_2_O_2_ as a substrate.

#### 2.4.2. Indirect Enzyme-Linked Immunosorbent Assay (ELISA)

ELISA plates were coated with purified PvTRAg (600 ng/well) suspended in 100 mM carbonate-bicarbonate buffer (pH 9.6) and incubated overnight at 4 °C. The plates were then washed using 200 µL of 1× phosphate buffer saline (PBS, pH 7.2) twice and blocked with 3% bovine serum albumin (BSA) in 1× phosphate buffer saline with 0.5% Tween 20 (1× PBST) for one h at 37 °C. After three washes using 200 µL of 1× PBST, the anti-PvTRAg sera (1:60,000) dilution was made in 1× PBST with 1% BSA. The plate was incubated for one h at 37 °C under static conditions. The plate was washed three times using 200 µL of 1× PBST to remove non-specific primary antibodies from polyclonal rabbit sera. The bound antibody was detected using goat anti-rabbit IgG-horseradish peroxidase conjugate (1:3000) in 1× PBST in 2% BSA. The plate was incubated at 37 °C under static conditions for one h. The unbound secondary antibody was washed using three rounds of 200 µL of 1× TBST. The interactions were developed using TMB/H_2_O_2_ as the enzyme substrate. A total of 20 µL of TMB/H_2_O_2_ was added to 20 mL of 1× PBS, then 100 µL of the solution was added to each well. The plate was incubated at RT for 8 min in the dark condition. The reaction was stopped by adding 50 µL of 2 M sulfuric acid, and optical density (OD) was measured at 450 nm using a multi-well plate reader (FLUOstar Optima, B.M.G. Labtech, Ortenberg, Germany).

### 2.5. Western Blot Using Rabbit Polyclonal Antibody and Purified PvTRAg along with Clinical Samples for Testing the Generated Antibody

The parasite pellet, *Plasmodium vivax*, was isolated using the saponin method from the vivax-infected patient’s whole blood sample and prepared the parasite lysate using a lysis buffer with a pH of 7.2 [20]. SDS-PAGE was run with 2 µL of blue ultra-pre-stained protein ladder, 2.5 µg of purified PvTRAg, and 5 µg of parasite lysate containing native PvTRAg. The SDS-PAGE was run in duplicate, one of which was used for Western blot transfer, and the other was used for Coomassie blue staining.

For Western blot transfer, the PVDF membrane was activated by incubating the membrane in 100% methanol for 5 min in rocking conditions, followed by incubation of the PVDF membrane, tissue stacks, and SDS-PAGE in transfer buffer for 5 min. The protein bands were transferred to the PVDF membrane by setting up the tissue stacks and activating the PVDF membrane, SDS-PAGE, then tissue stacks. Transfer buffer (glycine 14.4 g, tris-chloride 2.03 g, and 200 mL methanol in 1-L deionized water) was used to keep the gel and membrane wet during the transfer. Blot transfer was done at 25 volts, 1 Ampere constant for 45 min using a semi-dry blot transfer machine. A total of 3% bovine serum albumin (BSA, Sigma Aldrich) in 1× TBST (sodium chloride 8.5 g, tris-chloride 3.02 g, and 10 mL of Tween 20 in 1 L of deionized water) was used as a blocking agent on the transferred PVDF membrane. The membrane was incubated for 2 h for blocking at RT on the rocker at 20 rpm. After 2 h, the blocking buffer was discarded, and the membrane was washed thrice, using 1× TBST at RT on the rocker, at 50 rpm, for 10 min. The washed membrane was then incubated in 1:3000 ratios of vivax-infected plasma samples and 1% BSA in 1× TBST solution at 4 °C overnight on the rocker at 20 rpm. The PVDF membrane was then washed thrice using 1× TBST at RT on the rocker, at 50 rpm, for 10 min. The washed membrane was then incubated in a 1:5000 ratio of anti-IgG Fc region antibody (goat anti-human IgG (H + L) secondary antibody HRP, Invitrogen) conjugated with horseradish peroxide (HRP) in 1% BSA freshly prepared in 1× TBST. The membrane was incubated for 2 h in secondary antibody dilution at RT on the rocker at 20 rpm. The membrane was rewashed to remove the excess secondary antibody, using 1× TBST, thrice for 10 min at RT, the rocker condition was 50 rpm. The blot was developed using 20 µL of 3,3′,5,5′-Tetramethylbenzidine (TMB) in 10 mL of deionized water. The signals were read using a ChemiDoc instrument.

### 2.6. Development of Immune Assay Using PvTRAg and Synthesized Polyclonal Antibody for P. vivax Diagnosis

#### 2.6.1. Indirect ELISA Using Purified PvTRAg Protein to Identify Natural anti-PvTRAg Antibodies in the Host

Indirect ELISA was performed using 96-well plates (Thermo, maxisorp Nunc plate, Waltham, MA, USA). ELISA plates were coated with purified PvTRAg (600 ng/well) suspended in 100 mM carbonate-bicarbonate buffer (pH 7.2) and incubated overnight at 4 °C. The plate also has two negative control (200 µL Milli-Q water), two blank wells (200 µL coating buffer), and two wells with 600 ng tetanus antigen-coated with the same coating buffer as a positive control for ELISA. The plates were washed using 200 µL of 1× phosphate buffer saline (PBS, pH 7.2) twice and blocked with 3% BSA in 1× PBST for one h at 37 °C. After three washes using 200 µL of 1× PBST, non-severe vivax malaria (NSVM; *n* = 4), severe vivax malaria (SVM; *n* = 4), healthy control (HC; *n* = 3), non-severe falciparum malaria (NSFM; *n* = 2), severe falciparum malaria (SFM, *n* = 2), and dengue fever (DF; *n* = 2) plasma dilutions (1:150 and 1:200) were made in 1× PBST with 1% BSA. Each sample and its dilutions were plated in duplicates. The plate was incubated for 1 h at 37 °C under static conditions. The plate was washed three times using 200 µL of 1× PBST to remove non-specific primary antibodies from human plasma. The bound antibody was detected using goat anti-human IgG-horseradish peroxidase conjugate (1:3000) in 1× PBST in 2% BSA. The plate was incubated at 37 °C under static conditions for 1 h. The unbound secondary antibody was washed using three rounds of 200 µL of 1× TBST. The interactions were developed using TMB/H_2_O_2_ as the enzyme substrate. A total of 20 µL of TMB/H_2_O_2_ was added to 20 mL of 1× PBS, from which 100 µL of the solution was added in each well. The plate was incubated at RT for 8 min in a dark condition. The reaction was stopped by adding 50 µL of 0.2 M sulfuric acid, and optical density (OD) was measured at 450 nm using a multi-well plate reader, Multiskan GO (Thermo Fisher Scientific, Waltham, Massachusetts, United States cat. no. N10588).

#### 2.6.2. Antigen Capturing Using Biolayer Interferometry System for Diagnostics

The rabbit polyclonal antibody was biotinylated using EZ—Link N.H.S.—LC-LC—Biotin by adding 1 µg of purified rabbit polyclonal antibody and biotin in a 1:3 molar ratio. We mixed both reagents well in a polymerase chain reaction (PCR) tube and incubated the tube at RT for 45 min under shaking conditions at 200 rpm. After biotinylation, the mix was desalted using Zeba™ spin desalting columns, 7K molecular weight cut-off (MWCO; 0.5 mL, catalog number: 89882). The mixture was diluted to make the volume 100 µL by adding 1× PBS, it wasmixed well, and the solution was added to the Zeba column. The mixture was passed through the column by centrifuging at 2000× *g* for 2 min. The elute contained a biotinylated rabbit polyclonal antibody, and the Zeba column retained the unbound biotin. The column was then washed using five-bed volumes of 1× PBS and stored at 4 °C for future reuse. The biotinylated antibody was diluted in 1× kinetic buffer (provided by B.L.I., Sartorious). A total of 200 µL of this solution was used to immobilize antibodies on the streptavidin sensors for 10 min at 1000 rpm shaking.

The biotinylated antibody immobilized on the streptavidin sensors was used to capture purified PvTRAg in 7 different dilutions starting from 1 µM and diluted six times in a 1:1 ratio with 1× PBS. Signal capturing for the PvTRAg standards was used for standard curve preparation, which was used for absolute quantification of the free native PvTRAg captured from the diluted patient plasma (1:150 dilution) sample. The immobilized sensors were equilibrated in 1× PBS for 120 s. Free native PvTRAg capturing from diluted plasma sample was given 300 s, followed by three cycles of regeneration and neutralization for 5 s each. The whole experiment was performed at 1000 rpm using BLI (Sartorius)

## 3. Results

### 3.1. Polyclonal Antibody Generation against PvTRAg for the Development of a Diagnostic Assay for P. vivax

#### 3.1.1. Immunization and Titer Value

A test bleed was collected after the first booster from the marginal ear vein to check the level of circulating antigen-specific antibodies in the sera of the immunized rabbit. The presence of anti-PvTRAg antibodies was checked by indirect ELISA using purified PvTRAg (1 µg/well) as coating protein and 10 dilutions of rabbit sera starting from a 1:100 dilution in a 1:1 ratio for serial dilution. The antiserum obtained from the immunized rabbit had anti-PvTRAg antibodies detected by the ELISA assay. However, the antibody titer was not high in the first dose; hence another booster dose was given to check if there were any further improvements in the titer value (Figure 1b). The antibody titer experiment was repeated after seven days past the booster dose day. The protocol used for the sera obtained from the second bleed was the same as that of the first, except twelve dilutions of rabbit polyclonal antibody were used, starting from a 1:1000 dilution in a 1:1 ratio for serial dilution as the primary antibody. In the second bleed, an optimal dilution of 1:64,000 antibody to 1× PBST worked well for antigen and antibody interaction in indirect ELISA [24] (Figure 1c).

#### 3.1.2. Purification and Characterization of Rabbit Polyclonal Antibody

The purification of rabbit polyclonal antibodies was done using protein A affinity chromatography. After the chromatographic experiment, the purity of the rabbit polyclonal antibody was checked on SDS-PAGE gel stained with Coomassie blue (Figure 1d). It was found that the rabbit polyclonal antibody was purified to near homogeneity.

a. Western blot using purified PvTRAg and Rabbit polyclonal antibody

Western blot was correctly transferred, confirmed by Ponceau staining. The purified PvTRAg was run along with pure IgG as a positive control, and both the lanes gave good bands, proving the success of the antigen–antibody interaction (Figure 1e).

b. Indirect enzyme-linked immunosorbent assay (ELISA)

Indirect ELISA also confirmed the interaction of purified PvTRAg (600 ng/well) and a ratio of 1:64,000 rabbit polyclonal antibody to 1× PBS to be sufficiently apt to perform future experiments using the patient’s plasma samples (Figure 1b).

### 3.2. Western Blot Using Rabbit Polyclonal Antibody and Purified PvTRAg along with Clinical Samples for Testing the Generated Antibody

**Figure 2:**
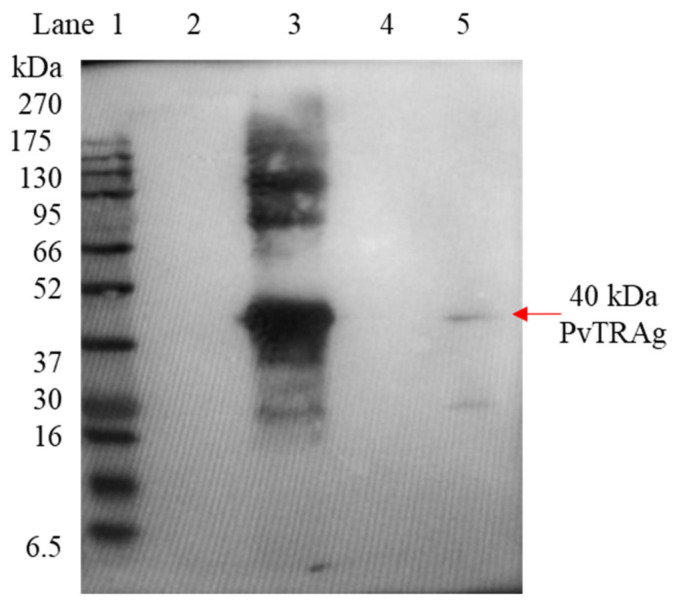
The Western blot used to identify the interaction between the native form of PvTRAg and synthesized Rabbit polyclonal antibody, where lane 1 is blue ultra prestained protein ladder, lane 2 is empty, lane 3 is 2.5 µg purified PvTRAg protein, lane 4 is empty, and lane 5 is 5 µg of parasite lysate.

The reactivity of the rabbit polyclonal antibody with the purified PvTRAg was analyzed by immunoblotting. The immunoblot showed that the rabbit polyclonal antibodies were specific towards PvTRAg as it recognized the recombinant purified PvTRAg protein. A clear band was observed in the parasite lysate lane at the same size where purified PvTRAg was found (Figure 2).

### 3.3. Development of Immune Assay Using Purified PvTRAg and Synthesized Polyclonal Antibody for P. vivax Diagnosis

#### 3.3.1. Indirect ELISA Using Purified PvTRAg Protein to Identify Natural anti-PvTRAg Antibodies in the Host

**Figure 3:**
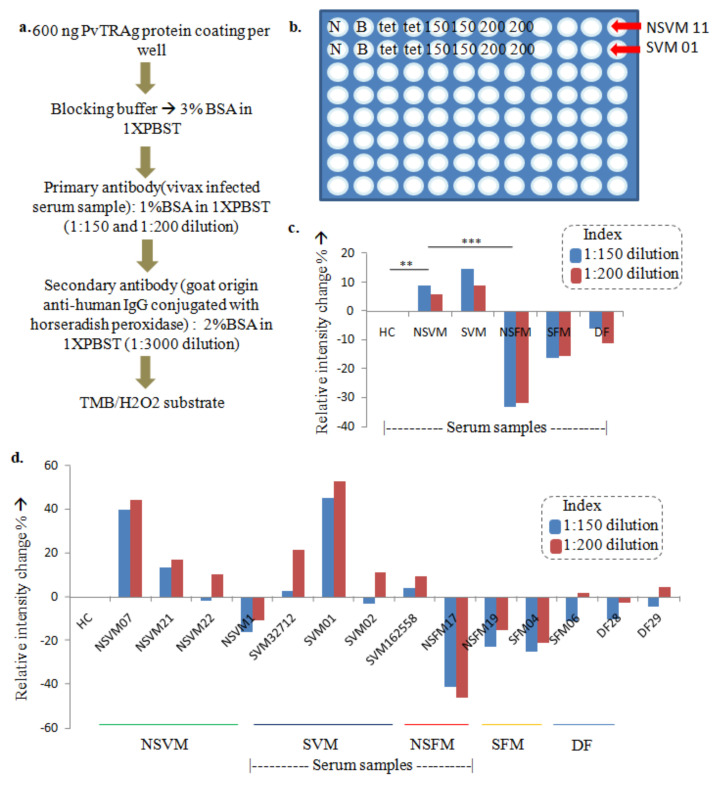
Indirect ELISA for monitoring the presence of natural anti-PvTRAg antibodies in healthy control (HC, *n* = 3), non-severe falciparum malaria (NSFM, *n* = 2), non-severe vivax malaria (NSVM, *n* = 4), severe falciparum malaria (SFM, *n* = 2), severe vivax malaria (SVM, *n* = 4), and dengue fever (DF, *n* = 2). (**a**) Workflow of indirect ELISA using PvTRAg, serum, and anti-human IgG. (**b**) Representative diagram of ELISA plate outline for negative control (N), positive control (tet–tetanus, 600 ng/well), and buffer (B) wells along with test samples (1:150 and 1:200 dilutions of different plasma samples). (**c**) Relative intensity percentage of the antigen–antibody interactions for the different sample types as compared to an average of the healthy patient plasma intensities (where ** represents difference between healthy and diseased samples > 5%, *** represents difference between healthy and diseased samples > 10%). (**d**) Relative intensity percentage of the antigen–antibody interactions for the different sample types compared to an average of the healthy patient plasma intensities.

The absorbance obtained through the antigen–antibody interactions for non-severe vivax malaria, NSVM (*n* = 4), severe vivax malaria, SVM (*n* = 4), healthy controls, HC (*n* = 3), non-severe falciparum malaria, NSFM (*n* = 2), severe falciparum malaria, SFM (*n* = 2), and dengue fever, DF (*n =* 2) plasma dilutions were analyzed. A sequential increase in the antibody reaction from HC to NSVM to SVM plasma samples was observed. The signals were found to be lowest in NSFM, followed by SFM and DF (Figure 3). The observation from the experiment suggests the absence or low binding of the antigen to the targeting antibodies making PvTRAg a reliable and potential diagnostic candidate.

#### 3.3.2. Antigen Capturing Using Biolayer Interferometry System for Diagnostics

**Figure 4:**
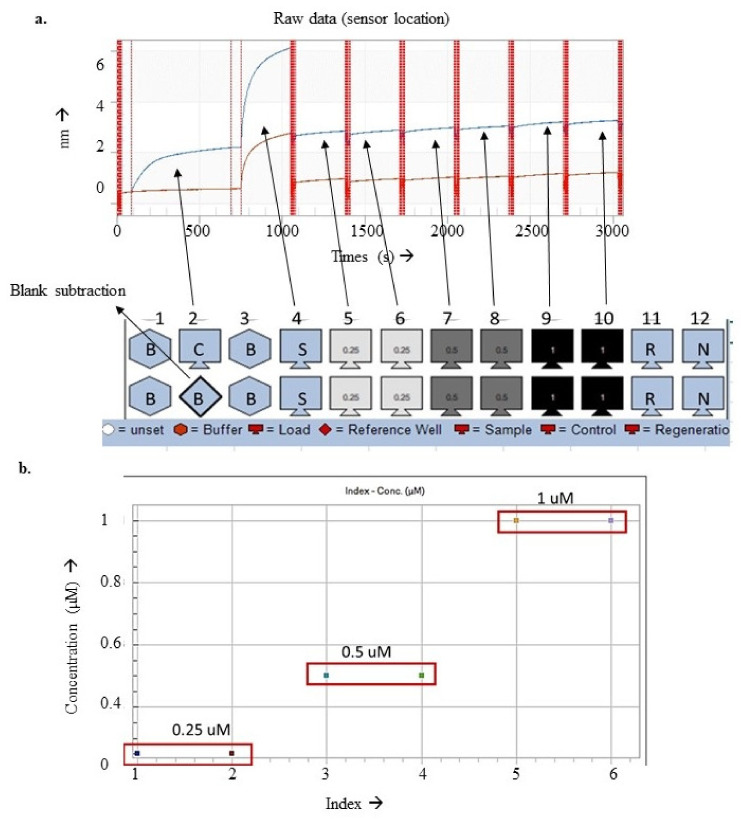
(**a**) Signal sequence of the capturing antibody (C) or buffer (B) plasma sample in a 1:150 dilution (S), standards in duplicates from 0.25 µM to 1 µM of purified PvTRAg. (**b**) Index vs. concentration plot to show the binding signals concerning the concentrations of the standard present in respective wells.

The biotinylated antibodies were immobilized to attain the saturation curve for 10 min (Figure 4a). The antibody-to-target interaction was observed maximum in the plasma sample (1:150 dilution), and a consistent signal increase directly proportional to the standards’ µM concentration was observed (Figure 4b).

## 4. Discussion and Conclusions

Parasite proteins from the PvTRAg family are reported to have a tryptophan-rich domain capable of generating an immune response in the infected host. The protein was also abundant in the parasite and plasma proteome studies [20]. The purified antibody and antigen pair can be explored and validated further for diagnostic purposes. The diagnostic candidate will facilitate the differentiation of vivax from the rest of the febrile disease pathogens.

The two possible ways to conduct a diagnostic immunoassay are (a) confirm the presence of antibodies against the pathogen protein; (b) confirm the presence of antigen of the pathogen. Out of the two cases, the latter is the more reliable and real-time status of the patient’s health. The former method may lead to false positives due to delayed clearance of the antibodies from the host system even after the infection is long gone. Therefore, a protein unique to *P. vivax* and abundant in parasite pellet and infected host plasma was chosen. Purified PvTRAg was used to detect anti-PvTRAg antibodies in patient samples. Individual samples’ ELISA intensity values represent possible biological variation; hence, the average shows the trend of antibodies in different clinical conditions. BLI helped in the possible detection of free native PvTRAg in a 1:150 diluted plasma sample. The absorbance shown by the plasma sample was out of range of the purified PvTRAg taken to prepare a standard curve. Hence, the assay needs further optimization by increasing the plasma samples’ normal curve range or dilution. However, high dilutions of plasma may lead to missed or no target protein identification as parasite proteins are low abundant proteins compared to the host proteins.

## Data Availability

Not applicable.

## Supplementary Materials

All the required information is present in the manuscript. The following supporting information can be downloaded at: https://www.mdpi.com/article/10.3390/diagnostics13050835/s1, Figure S1: Diagram represents parasite proteins reported repeatedly in all studies on clinical samples.
