# Supplementary material for: Polyclonal Antibody Generation against PvTRAg for the Development of a Diagnostic Assay for Plasmodium vivax
Source: Diagnostics (Basel). 2023 Feb 22;13(5):835. doi: 10.3390/diagnostics13050835 (PMC10001162; doi:10.3390/diagnostics13050835)
Supplement: Supplementary file 1 [file diagnostics-13-00835-s001.zip › diagnostics-2061024-supplementary.pdf]

Article

Polyclonal Antibody Generation against PvTRAg for the Development of a Diagnostic Assay for *Plasmodium vivax*

Shalini Aggarwal [1,2], Selvamano Selvaraj [3], Jayaprakash Nattamai Subramanian [3], Mookambeswaran Arunachalam Vijayalakshmi [3], Swati Patankar [1] and Sanjeeva Srivastava [1,*]

[1] Department of Biosciences and Bioengineering, Indian Institute of Technology Bombay, Mumbai 400076, India
[2] Department of Molecular Genetics, Weizmann Institute of Science, Rehovot 7610010, Israel
[3] Centre for Bio-Separation Technology, Vellore Institute of Technology, Vellore 632014, India
* Correspondence: sanjeeva@iitb.ac.in; Tel.: +91-(22)-2576-7779

Supplementary data

A. The ELISA was performed with preimmunized Rabbit serum and immunized rabbit serum (after booster dose) and the absorbance data found was

SAMPLE	Primary Antibody Dilution (1:)	OD @450nm	
		O.D. 1	O.D. 2
Blank	0	0.084	0.064
Pre-Immunized Rabbit serum	2000	0.118	0.109
Immunized Rabbit serum	2000	3.326	3.301

B. Inclusion and exclusion criteria for clinical samples used in the study are –

Inclusion criteria (Healthy Controls):

1. Participants of either sex, between 18-65 years of age.
2. No recent history of febrile illness or other disease conditions.
3. Those willing to provide written informed consent and comply with protocol requirements

Inclusion criteria (Malaria):

1. Participants of either sex, between 18-65 years of age.
2. Patients with *P. vivax* or P. falciparum infection confirmed with microscopic examination and/or RDT
3. Those willing to provide written informed consent and comply with protocol requirements

Exclusion criteria (Malaria and Healthy individuals):

1 People with a history of significant systemic diseases like autoimmune disorders, chronic liver disease, chronic kidney disease, neuro-psychiatric illness, or any other illness likely to affect blood biochemistry/ cytology, as judged by history and physical examination.

2. Co-infection with dengue, chikungunya, viral hepatitis, typhoid or any other commonly prevalent infectious disease

3. Subjects unwilling to consent to the study.

Supplementary Figure S1:

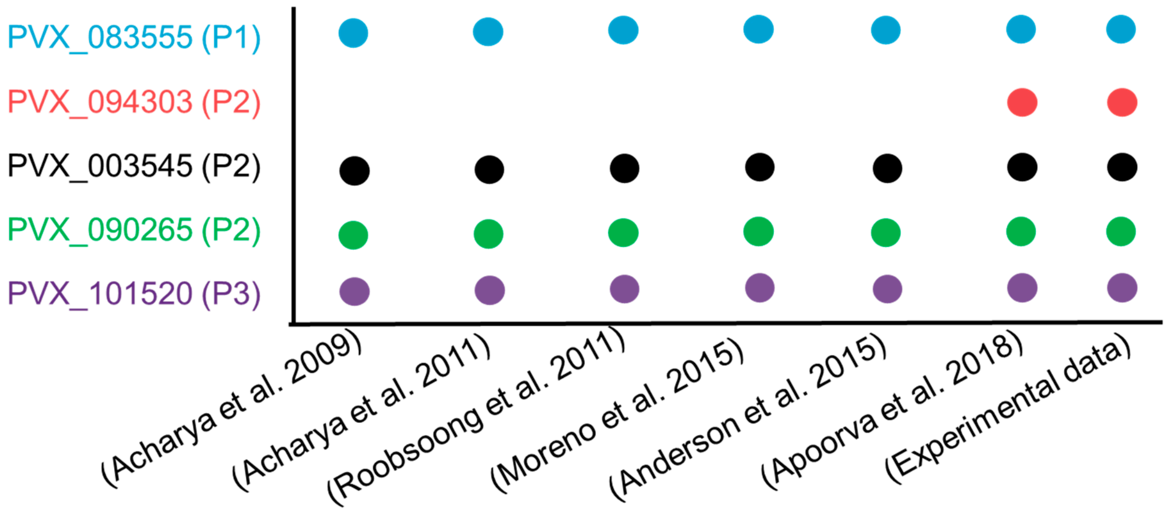

Figure S1: Diagram represents parasite proteins reported repeatedly in all studies on clinical samples.
